# Supplementary material for: Exploring Patient Views and Acceptance of Multiparametric Magnetic Resonance Imaging for the Investigation of Suspected Prostate Cancer (the PACT Study): A Mixed-Methods Study Protocol
Source: Methods Protoc. 2020 Mar 28;3(2):26. doi: 10.3390/mps3020026 (PMC7359448; doi:10.3390/mps3020026)
Supplement: Supplementary file 1 [file mps-03-00026-s001.pdf]

Supplementary Material

Supplementary Document 1

Acceptability of Prostate MRI:

Patient Consent Form 1 (Questionnaire)

Please initial box

1. I confirm that I have read and understood the information sheet dated ………… (version …………) for the above questionnaire study. I have had the opportunity to consider the information, ask questions and had these answered satisfactorily. ☐

2. I understand that my participation is voluntary and that I am free to withdraw at any time without giving any reason, without my medical care or legal rights being affected. ☐

3. I agree to my GP being informed of my participation in the study. ☐

4. I agree to take part in the above study. ☐

Name of participant:

Date:

Signature:

Name of person taking consent:

Date:

Signature:

Supplementary Document 2

MRC Medical Research Council | UCL | University College London Hospitals NHS Foundation Trust | Whittington Health NHS Trust

Acceptability of Prostate MRI:

Patient Information Sheet

- In the past (1980s – 2000s) – MRI had very limited availability and was not commonly used:
    - Men suspected to have prostate cancer would not normally have an MRI scan
    - They would normally undergo a prostate biopsy (tissue sample) through the rectum (bottom)
    - This could cause side-effects, including: pain, infection (1-3%) and bleeding

> But, this technique did not detect every prostate cancer: approximately 50% could still be missed
>
> - Furthermore, many of these men did not need a prostate biopsy in the first place

- Today – MRI is now widely available in the UK:
    - We now use MRI scans to see if prostate cancer is likely to be present or not (before a biopsy is performed)
    - If the MRI scan shows:
        - Suspicious areas in the prostate – then you will likely be advised to still have a prostate biopsy
        - No suspicious areas in the prostate – then you will probably not need to have biopsy
    - MRI scans can now detect around 80-90% of important prostate cancers
    - So, by using MRI we can prevent many men from unnecessarily having a prostate biopsy

> But, MRI does not detect every prostate cancer: approximately 10-20% can still be missed by MRI
>
> - Therefore, by using MRI, we could miss approximately 10-20% of important prostate cancers

Supplementary Document 3

Acceptability of Prostate MRI:

Patient Questionnaire

(*Adapted from: Schönenberger et al. 2007, with kind permission of Professor Marc Dewey*)

Please give your answers based on your own knowledge and having read the patient information sheet.

---

1. How satisfied are you with the ability of prostate biopsy alone to help detect prostate cancer?

   Very poor ☐ Poor ☐ Barely Acceptable ☐ Good ☐ Very good ☐

2. How satisfied are you with the ability of MRI scans to help detect prostate cancer?

   Very poor ☐ Poor ☐ Barely Acceptable ☐ Good ☐ Very good ☐

---

3. Please rate your degree of concern that your MRI scan might miss important prostate cancer:

   No concern ☐ Little ☐ Moderate ☐ Intense ☐ Very intense ☐

   If you are concerned, why are you concerned? …………………………………………………………………………...

---

4. If your MRI scan showed low suspicion of prostate cancer, would you be happy to not have a prostate biopsy?

   No ☐ Yes ☐ Don't know ☐

5. If your MRI scan showed low suspicion of prostate cancer, would you want to have a biopsy anyway?

   No ☐ Yes ☐ Don't know ☐

---

6. If you were diagnosed with prostate cancer, which aspect of the disease do you think would be most important to you? (For example: life expectancy, quality of life, spread of cancer around the body, urine/sexual symptoms)

7. Are there any additional comments that you would like to make?

If you would be willing have a short interview on this topic, then please leave your contact details below:

Phone number:
Email address:
Postal address:

Supplementary Document 4

Acceptability of Prostate MRI:

Patient Consent Form 2 (Interview)

Please initial box

1. I confirm that I have read and understood the information sheet dated ………… (version …………) for the above interview study. I have had the opportunity to consider the information, ask questions and had these answered satisfactorily. ☐

2. I understand that my participation is voluntary and that I am free to withdraw at any time without giving any reason, without my medical care or legal rights being affected. ☐

3. I agree to my GP being informed of my participation in the study. ☐

4. I agree to take part in the above study. ☐

Name of participant:

Date:

Signature:

Name of person taking consent:

Date:

Signature:
